# NIPT Integration as a Patient-Paid Prenatal Screening Option—Observations and Challenges from a Bulgarian Genetic Counseling Center

**DOI:** 10.3390/medsci13010003

**Published:** 2024-12-29

**Authors:** Dinnar Yahya, Mari Hachmeriyan, Milena Stoyanova, Mariya Levkova

**Affiliations:** 1 Department of Medical Genetics, Faculty of Medicine, Medical University of Varna, 9002 Varna, Bulgaria; mari.hachmeryan@mu-varna.bg (M.H.); milena.stoyanova@mu-varna.bg (M.S.); mariya.levkova@mu-varna.bg (M.L.); 2 Laboratory of Medical Genetics, UMHAT St. Marina, Hristo Smirnenski blv 1, 9000 Varna, Bulgaria

**Keywords:** NIPT, genetic counseling, prenatal diagnosis, genetic testing

## Abstract

*Background*: NIPT is a widely implemented method for prenatal screening of chromosomal disorders. Its introduction initiated the practice of counseling women pre- and post-analytically. Since the test’s usage is established in different conditions, comparing data from various socioeconomic and cultural backgrounds would be of scientific value. Our study is the first to describe NIPT integration in Bulgaria. We aimed to evaluate current trends in demand and referral, the frequency of high-risk results, cases of fetal sex discrepancies and their impacts, as well as commonly held misconceptions during genetic counseling. We also address issues and necessary general prophylaxis and prenatal care improvements. *Methods*: We performed a retrospective analysis on the pregnant women who received GC for NIPT in our genetic center between 2016 and 2023. We separated this period into two due to a significant difference in the test’s price. A total of 635 women were included with their referral indications, panel width preference, fetal sex, and SCA. We assessed cases of fetal sex discrepancy, high-risk pregnancies, late NIPT (after GW 18), and commonly occurring issues and misconceptions. *Results*: We observed a significant increase in the demand for NIPT—63 women for 2016–2020 versus 572 for 2021–2023. The leading indications were supervision of normal pregnancy (50.4%) and advanced maternal age (>35 years) (31.2%). As for late NIPT, the most common indications for this late testing were high risk from a maternal serum screening test (33.3%) and anxiety (25%). Further, 1.1% of results were high-risk for trisomy 18 and 21 and monosomy X. We reviewed two cases of fetal sex discrepancy (0.3%) and common misconceptions twice more during pre-test GC. *Conclusions*: This single-center experience shows that demand for NIPT is exponentially growing, especially as a normal pregnancy screening method. Delivering thorough education to the community and guaranteeing outstanding care during genetic counseling sessions is crucial for fostering informed decisions and overall well-being.

## 1. Introduction

The swift advancement of modern molecular technologies has prompted a shift towards non-invasive techniques across various medical fields. This tendency has also affected prenatal care, with the possibility of evaluating fetal risk for genetic disorders. Thanks to its well-established effectiveness and decreasing costs, non-invasive prenatal testing (NIPT) is increasingly preferred for screening chromosomal conditions such as trisomy 21, 18, and 13. Slowly yet steadily, the test has replaced the less precise maternal serum screening method and provides better filtering for patients worldwide who need an invasive diagnosis [1]. NIPT’s introduction also initiated the practice of counseling women pre- and post-analytically. In Bulgaria, this is not strictly organized and is partially covered by physicians who are either obstetricians and gynecologists (OBGs) or medical geneticists. The latter provides genetic counseling (GC) since the separate profession of a genetic counselor does not yet exist in our healthcare system. The advance in prenatal screening generates a need for specialist care with a deep and thorough understanding of NIPT’s potential, limitations, and probable impact on the pregnancy and the family, given their cultural, educational, and socio-economic background. Its wide time window and additional possibilities, like screening for fetal sex, sex chromosomal aneuploidies (SCAs), rare autosomal anomalies (RATs), big structural changes, and even microdeletions and microduplication detection, are further complicating women’s choices and therefore the need for counseling. Additionally, the sensitivity and specificity limitations for SCA and rarer chromosomal changes are sometimes disregarded when opting for a more comprehensive panel. These and other characteristics of NIPT generate several ethical foci that demand clarification by genetic counselors [2,3,4,5,6,7].

Since its introduction in 2011 [8], the test has been offered as first-tier, second-tier, or optional in different countries, depending on national healthcare policies and coverage [9,10,11,12]. Researchers’ experiences with NIPT implementation recurringly emphasize the need for thorough and personalized genetic counseling. Issues concerning the test’s scope, specificity, sensitivity, consequences for the pregnancy depending on the result, and common misconceptions have been discussed in different cultural and socio-economic settings.

We aim to share our experience and perspective on GC for NIPT regarding the contingency of patients, considerations, challenges, trends noticed, and conclusions from our 8-year implementation. Our center is based in Eastern Europe, where maternal serum screening, including first and second trimester in combination with ultrasound (US), is free to all women in the country. Meanwhile, NIPT is available as a patient-paid option for all pregnant patients [11]. Both the provider and panel width of the test can be chosen. Although we realize that finances are a biasing factor, we still find our insights relevant, as they describe an ongoing situation in economically developing countries. To the best of our knowledge, this is the first study to describe the period of introduction of this advanced screening method as a patient-paid system in our country. Thus, cultural and economic differences could create variations in contrast to other countries and systems. We hope our data can be used for comparison and further analysis in future studies.

## 2. Materials and Methods

We performed a retrospective analysis on the contingency of pregnant women who underwent NIPT and received pre- and post-test GC in the Laboratory of Medical Genetics, University Hospital “Sveta Marina”, Varna, for eight years—from January 2016 to December 2023. We established inclusion criteria, where all must be met. We assessed all women with a confirmed pregnancy by an OBG with a recently performed US who could undergo the sample collection, were financially able to cover the test, and signed an informed consent about NIPT. Our exclusion criteria were lack of pregnancy confirmation with the US, rejection or inability to participate in sample collection, inability for patient payment, and lack of informed consent. One criterion would be enough to exclude the participant from our study. We further separated the one period into two—2016–2020 and 2021–2023—due to a significant difference in the test’s price. This difference was about 2–3 times, with the earlier option costing about EUR 600–800 and the newer EUR 200–300, depending on the chosen panel. For financial reference, in 2016, the minimum monthly salary in Bulgaria was EUR 214, while the average was EUR 480 [13]. In 2021, these numbers increased significantly to EUR 332and EUR 888, respectively [14]. Regarding the variety of tests offered, all providers suggest several options—trisomy 13, 18, and 21, with or without sex and sex chromosomal aneuploidies. A more expansive option exists, assessing all chromosomes for changes more significant in size than 7 million base pairs. Only one of the providers included microdeletion syndromes in its most expansive panel. However, its services were discontinued shortly after being introduced due to the USA-based company’s decision to cease market to Europe. We included 635 women aged 19–46, with a median of 33 years. We assessed their referral indications and the preference for the width of the panel regarding fetal sex and SCA. To eliminate financial limitations in decision-making, we also selected 44 women who underwent NIPT at a consistent price for all available panels. This pricing option was offered exclusively by one provider for a limited time. We separately assessed all high-risk results and the potential outcomes in such cases. Common issues and misconceptions during all GC sessions were also outlined and investigated. To assess the women looking for a “late” NIPT, we accepted a threshold of >18 gestational weeks (GW) per turnaround time and the possibility of providing a prenatal diagnosis after it (until 20th–21st GW for amniocentesis). The cases of fetal sex discrepancies between NIPT and ultrasound (US) findings that we encountered and their follow-up were also analyzed. All samples were processed by foreign outsourcing companies (in the USA, Germany, and France) based on women’s preference for the accessible ones. NIPT was only offered as a patient-paid option and as an alternative to or following the free-of-charge maternal serum screening. Data analysis was performed using Microsoft Excel v.16.0 and GraphPad Prism v. 9.5.1. We accepted a statistical significance level of *p* < 0.05.

## 3. Results

### 3.1. General Demand and Referral Indications

**Figure 1:**
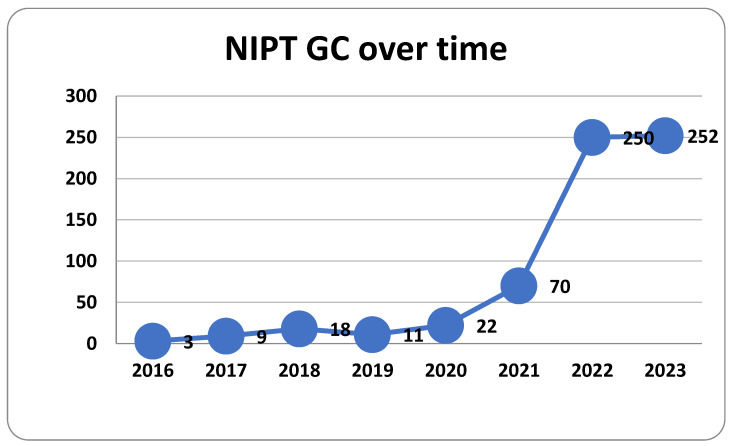
NIPT GC over time.

There was a remarkable rise in the demand for NIPT after 2021—63 women for 2016–2020 versus 572 for 2021–2023 (*p* < 0000.1, chi-square test) (Figure 1).

**Figure 2:**
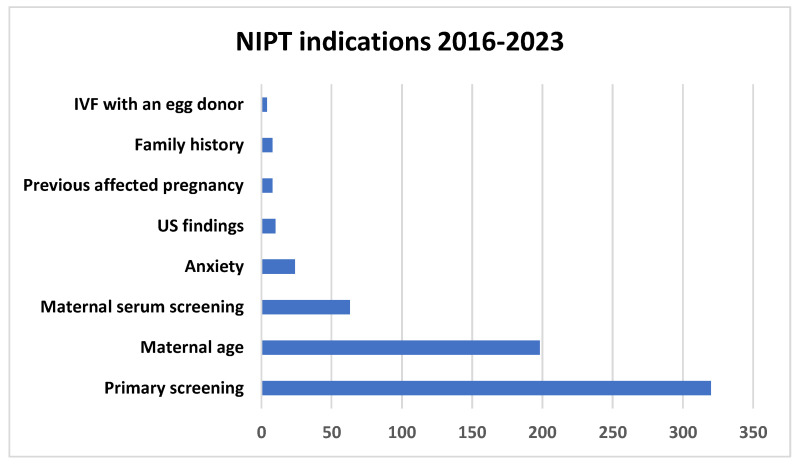
NIPT indications over time.

As for indications, the leading ones were supervision of normal pregnancy (50.4%) and advanced maternal age (>35 years) (31.2%). Positive maternal serum screening ranked third with 9.9% (Figure 2).

**Figure 3:**
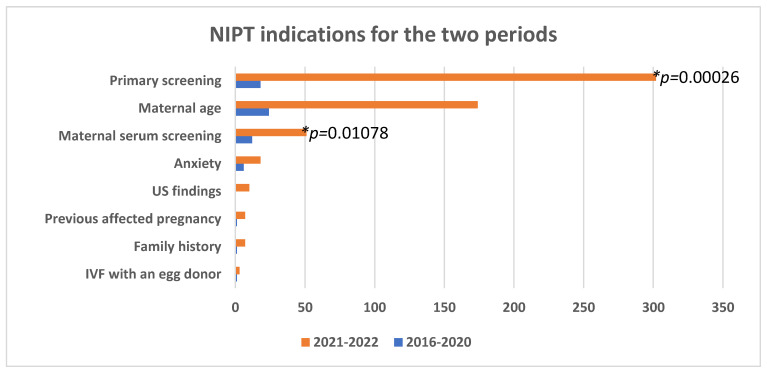
NIPT indications during the first and second period.

We compared the two periods to see if tendencies changed in time and with the decreased price for the test. During the first, more expensive period, women predominantly chose NIPT as a more sensitive test in cases of advanced maternal age (38.1%). Another 28.6% chose the test as a method for normal pregnancy follow-up. NIPT followed positive maternal serum screening in 19.1% of cases for this period. In 2021–2023, it was statistically significantly more commonly preferred for primary screening of normal pregnancy (52.8%) (*p* = 0.00026, chi-square test). Maternal age and positive serum screening represented 30.4% (*p* = 0.2113, chi-square test) and 8.9% (*p* = 0.01078, chi-square test), respectively (Figure 3). Of note, we labeled the indication as “Anxiety” in all cases of normal pregnancies where, regardless of young age (less than 35 years) and low risk from maternal serum screening, women still chose to undergo NIPT.

### 3.2. Expanded NIPT

**Figure 4:**
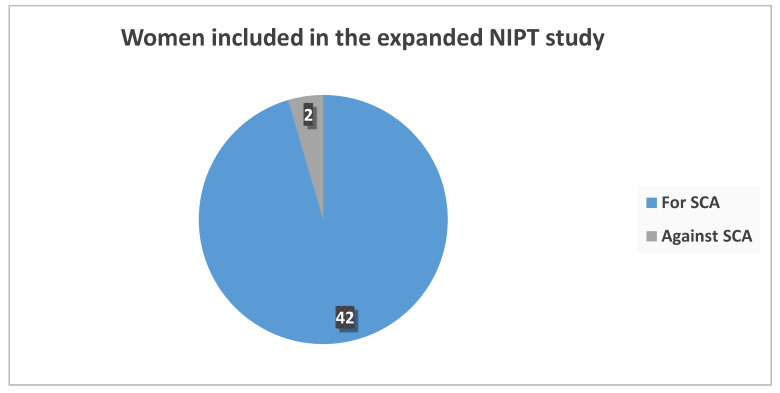
Women’s preference for NIPT’s panel width.

The selected group (*n* = 44) was offered NIPT with or without fetal sex and SCAs at the same price given the provider’s offer. The majority (95.5%, *n* = 42) preferred to include this information regardless of the indication for referral (Figure 4). Furthermore, all involved genetic counselors in the genetic center (*n* = 5); women, most of whom had one or more children before the age of 35, declared a personal preference for expanded NIPT. The latter information was not disclosed to the consulted women to preserve GC’s non-directive approach.

### 3.3. High-Risk from NIPT Pregnancies

We had seven high-risk results, 1.1% of all consulted pregnant patients. Of them, three were with increased risk for trisomy 18, two for trisomy 21, and two for Turner syndrome. We have information regarding outcomes in three cases: The two pregnancies at risk for trisomy 21 were referred for cytogenetic analysis in our center after amniocentesis. Both were confirmed with a complete free trisomy 21. Of note was the maternal age, 36 and 39 years in these cases. The third is a pregnancy with a high risk for Turner syndrome—a miscarriage occurred around the time of NIPT result availability. A further analysis of abortion material was not requested nor performed.

### 3.4. Late NIPT

**Figure 5:**
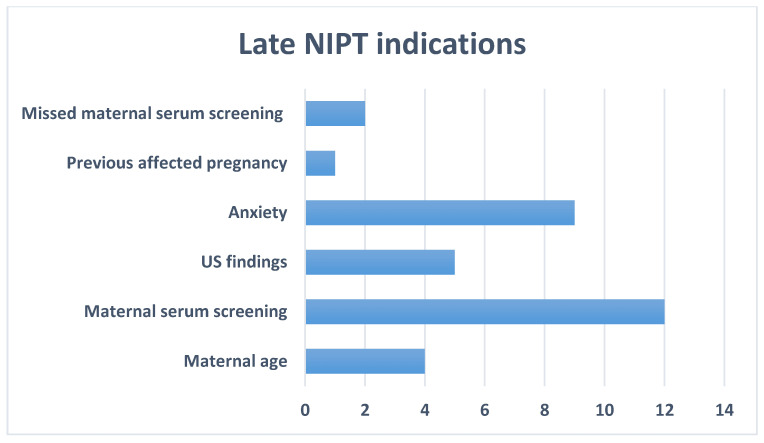
Late NIPT indications.

We evaluated 36 women (5.7% of all) between 2016 and 2023 who fit the criteria, the latest being in GW 27. The most common indications for this late testing were high risk from a maternal serum screening test (33.3%) and anxiety (25%) regarding the pregnancy. The latter indication matches the description above. Rarer cases were due to an abnormal US (13.9%), advanced maternal age (11.1%), missed early and late maternal serum screening (5.5%), and previous pregnancy with a chromosomal condition (2.8%) (Figure 5). As for the results, only one NIPT showed an increased risk for trisomy 21, while the rest were inconspicuous.

### 3.5. Sex Chromosome Discrepancies with US

We met two families (0.3%) for the entire period with such a discrepancy. Family 1 included a 39-year-old female with a reproductive history of one live-born child and an early miscarriage. The US performed at 14th GW before NIPT indicated uncertainty about the fetal sex; however, the specialist leaned toward it being a male fetus. NIPT results showed no Y chromosome, suggesting a female sex. The family expressed a great measure of anxiety during a following GC, where we suggested discussing additional steps if needed after the upcoming imaging study check. The subsequent US at the 16th week of gestation confirmed the NIPT result, as well-defined female genitalia were more visible at this later stage. We consulted the family with advice to reach us in case of continuing discrepancies. They have not contacted us since, so their privacy was respected.

Family 2: A 34-year-old female in her second pregnancy, with one live-born child, has an unremarkable family history. NIPT performed at 12 weeks showed the presence of a Y chromosome, while the subsequent US at 14 weeks indicated a female sex. Follow-up was ongoing because the second US had not yet been performed. Again, our approach was to consult the family and remain available to provide additional advice if needed. We were not contacted further.

## 4. Discussion

### 4.1. General Demand and Referral Indications

We noticed that the total number of women was unevenly spread for the evaluated period (Figure 1). We relate this difference to the popularization of the test and the introduction of a 2–3-times more affordable NIPT option. We think this process represents the growth of the community’s awareness resulting from self-driven health education and the information clinicians provide. The visible plateau in 2022–2023 of around 250 patients per year will be followed-up in the coming years to investigate further tendencies.

This test showed a notable shift in the reasons for using NIPT for routine pregnancy monitoring. As seen in Figure 3, the search for the method as a routine screening was nearly doubled—from 28.6% to 52.8%. We relate this tendency to the reduced test price and women being better informed in time. More and more of them realize that even at a young age, there is a risk for a sporadically occurring chromosomal disorder. Also, even after a normal result from maternal serum screening, some prefer undergoing NIPT, as it offers the reassurance of a superior, highly sensitive prenatal screening. Additionally, the introduction of a method that refines the need for an invasive procedure, such as amniocentesis, reduces pregnancy-related anxiety. We expect this tendency to develop in time and represent a more significant proportion of future studies. A potential transition into opting for NIPT as a primary screening method for all women would be beneficial since most pregnancies are categorized as normal and still carry a risk of resulting in a child with a chromosomal disorder. Until national healthcare systems include this method in routine coverage, financial costs will remain a significant limiting factor for pregnant women. Although its price was significantly reduced in time, we probably reached economically stable women and families only. Meanwhile, children born in less financially advantageous environments—about one in four are below the poverty mark in Bulgaria [15]—are left without such screening. In some countries, such as Japan, it is only available for women over 35 and only as a patient-paid option. In Australia, NIPT has been available since 2012 for all women with varying panel coverage and is also patient-paid. In other countries, such as the Netherlands, France, and the United Kingdom, it is provided to all women as a lower-priced option via patient and national healthcare co-payment [2,9,10,12]. Thus, an evident financial inequality exists among countries and different geographical regions within these countries. We recognize this bias in our study since it only includes women who could afford to undergo NIPT. Still, this is the integration model of this screening method in Bulgaria. Perhaps we could improve the robustness of our data in a future study that provides for women opting for a free-of-charge NIPT covered by national healthcare.

### 4.2. Expanded NIPT

There is an ongoing debate concerning diagnosing, treating, and counseling patients in prenatal testing care about SCA. Controversy remains regarding offering expanded NIPT to patients due to its limited sensitivity, specificity, and ethical implications. Cultural factors should be carefully taken into consideration to avoid practices such as fetal sex selection [16,17,18]. In our study, most women would likely choose expanded NIPT beyond the most common aneuploidies if financial constraints were removed. This tendency was expected based on previous studies [19,20]. It may correlate positively with factors arising from the counselor and the pregnant patient. Providing and regularly updating information about this method is crucial to enable individuals to make informed and independent decisions. It is noteworthy that our genetic counselors also preferred this option due to the high incidence of SCAs regardless of the reduced sensitivity and specificity of the test. Their choice provides an interesting perspective of being in both roles—a counselor and a patient—for a limited period. Their personal preferences remained undisclosed to the patients, as the non-directive approach is strictly followed in our center. Interestingly, genetic counselors frequently face questions regarding their preferences from patients puzzled by their options. We relate this need to the previously popular directive approach among all medical specialists in the country. Even though these questions reflect a much-needed trust in the counselor, we maintain the patients’ information-based independence in making such essential decisions.

### 4.3. High-Risk from NIPT Pregnancies

We identified a small number of high-risk cases: 1.1%, significantly lower than in the study from Gug et al. at 2.6% (*p* = 0.03318) [21]. Still, these cases must be separately addressed in practice, as they necessitate an individual approach. Since NIPT is a screening method, such results require genetic confirmation before discussing the possible pregnancy outcomes with the family. Due to its safety and the favorable time window, Bulgaria’s most common invasive procedure is amniocentesis, available between GW 15 and 21. It is free of charge for all high-risk pregnancies, including such results from maternal serum screening and NIPT. Also, unlike chorionic villous sampling (CVS), this is the recommended technique for high-risk NIPT result confirmation since it guarantees the testing of fetal and not placental cells [22]. Although CVS was an option in the capital city, we did not recommend it actively, and the pregnancy was usually already advanced. Similarly to other centers’ practices, the amniotic fluid is used for confirmation through cytogenetic analysis and polymerase chain reaction or microarray [21]. Other than the result from the confirmatory test, another aspect to be addressed during a third GC session would be the possible future of these pregnancies. While we cannot suggest any outcomes and decisions, the possible syndrome with its characteristics and prognosis has to be explained to the family. Thus, an informed choice can be secured. As for outcomes of high-risk pregnancies, there is a variety of them, depending on several factors. Some couples prefer to continue the pregnancy and use the opportunity to prepare for welcoming a child with special needs into their family. Providing information regarding patient groups other than treatment and monitoring regimens for these cases is essential. Others cannot cope with such a perspective and prefer to opt for termination of pregnancy (TOP) if the gestational week allows it. Although our study did not represent the population, most families shared this view. It is not prohibited in the country for medical reasons, but it is only possible before the 22nd GW and after confirmation of the result from the screening method. Thus, only cases before the 20th–21st week are applicable and require a fast approach. Others with very advanced pregnancies would only benefit from the opportunity to try and mentally prepare for having a child with a chromosomal disorder. Of course, this information is also invaluable for the medical team that will deliver the child and offer neonatal care.

### 4.4. Late NIPT

One of the known limitations of NIPT is the circulating free fetal fraction that requires performing it after the 10th gestational week [23]. However, late testing, performed near or after the latest possible time for invasive confirmatory testing, might also be problematic. In Bulgaria, amniocentesis is the most widespread and commonly preferred invasive technique for prenatal diagnosis, given its timing during pregnancy and its low risk for miscarriage [24]. Another issue would be the possible future of a late high-risk pregnancy. As discussed, outcomes depend on both families’ inclinations and the GW. Thus, only cases before the 20th week are applicable for confirmation and, eventually, TOP. Late NIPT was a relatively rare event in our center. The slow accumulation of such cases made us wonder about the tendencies in this small group, such as indications, panel choice, and results. As the significant indications for late NIPT do not differ significantly from the earlier ones (Figure 5), we may conclude that there is still a lack of widely available information about the possibilities of NIPT. Despite the scarceness of these cases, each one must be approached individually depending on the families’ indications, reproductive history, background, and cultural principles. Also, an explicit written recommendation for continuing pregnancy follow-up, including fetal morphology US, is fundamental.

### 4.5. Sex Chromosome Discrepancies with US

Since its introduction in 2011 [8], the commercialization of NIPT has significantly enhanced our ability to detect risks for chromosomal aneuploidies at an earlier stage [25]. While convenient, fetal sex identification may create difficult situations for parents and prenatal care providers [5]. The two cases represent a higher frequency than those in the significant study by Dhamankar et al., i.e., 0.007% (*p* < 0.00001), and the review by Smet et al. [26,27]. Although intriguing, this difference might be related to US performance, among other reasons, and is outside the scope of our study. Such discrepancies would understandably cause significant anxiety and stress for expecting parents. As a first step in both cases, we recommended a repeated US. Our first case was resolved since the second assessment aligned with NIPT results. However, ongoing follow-up care until and after the child is born is considered essential, even in such cases. Maternal contamination, different types of mosaicism, and the exclusion of vanishing twins may also be necessary to investigate if US findings continue to contradict the NIPT results. Postnatal cytogenetic analysis can be applied if suspicion of ambiguous or discrepant sex exists. Additionally, cases of potential disorders of sexual development (DSD) or other likely conditions should be confirmed through appropriate genetic analysis [26,27]. Since the classification of these disorders is immensely wide and diverse, careful clinical care must be implemented for the diagnosis, management, and monitoring of children with a confirmed DSD. As many also affect other systems and organs, the clinical approach must be multidisciplinary. Additional genetic counseling may be necessary for hereditary cases, along with ongoing psychological aid and participation in patient support groups.

### 4.6. Issues and Common Misconceptions During GC

We herein outline the most commonly reappearing misconceptions during pre- and post-test GC. They were twice greater more during the former. During pre-test GC, families were mainly confused about the following:

1. Expected mandatory inheritance of chromosomal syndromes and lack of such conditions in healthy families;
2. False reassurance in choosing the broadest panel available to assumingly include all possible genetic conditions;
3. False reassurance in being a young woman or couple, excluding the possibility of having a child with a chromosomal syndrome;
4. Expecting a healthy child due to already having one from a previous pregnancy;
5. Expecting the DNA test to be 100% accurate;
6. Thinking that being referred for NIPT by their OBG indicates an existing clinical suspicion regarding the pregnancy.

Regarding post-test GC, we mainly addressed some misconceptions about the following:

1. A normal result ensuring a healthy child;
2. The lack of need for regular health checks until birth in case of a normal result from NIPT;
3. An abnormal result guarantees an affected pregnancy.

Despite the significant increase in demand during the studied period, misconceptions among consultants persist. The main discussion topics were the origins of chromosomal aneuploidies, their heredity, the coverage of methods used to detect them, and the level of absolute reassurance one can expect. Unmet expectations during post-test counseling were lower since the essence of the conditions screened and the method used were thoroughly explained during the pre-test GC. This ongoing trend made us aware of potential feelings of disappointment, misunderstanding, and misjudgment among our patients. One of the main tasks of genetic counselors is to carefully address and explore these misconceptions, taking enough time and using simple language. Each counseling session would end with a resume of the most important information and clarification of any new questions. We also maintain contact with the consulted families until and after the results are available, with the option to meet and discuss issues and concerns. Still, a broader approach is probably needed to ensure satisfactory general knowledge regarding chromosomal conditions’ awareness at a populational level. This need is related to the functions of OBGs and genetic counselors in the healthcare system. We believe that providing accessible information nationwide will improve both general awareness and prenatal care effectiveness. Although this study cannot establish populational level approaches, we could suggest several possible steps to improve prenatal care:

1. Implementing or improving the existing physician training modules focusing on prenatal care. These would be essential for general practitioners, OBGs, pediatricians, and medical students;
2. Providing leaflets with general information regarding prenatal screening options. In clear language, they could include information regarding chromosomal conditions’ etiology and frequency, and screening methods (both free of charge and paid), and contact information of major providers, including genetic counselors. The leaflets could be located at pharmacies, OBG, and general practitioners’ offices to reach as many women as possible;
3. Implementing mass media tools to improve the general population’s health education and awareness—television, internet, social media, or radio—to reach as many people as possible;
4. Comprehensive health and sexual education in high schools can address general biological and sexual health principles, providing a solid foundation of knowledge. Most commonly detected misconceptions can be easily avoided.

## 5. Conclusions

Pregnancy involves numerous medical appointments and can bring emotional stress, requiring quick decision making. Since NIPT is not part of the Bulgarian prenatal care program, self-motivation, education, and referrals to specialists are essential for promoting this method. This single-center experience shows that demand for NIPT is exponentially growing, especially as a normal pregnancy screening method. High-quality care during genetic counseling sessions is essential for medical geneticists’ practice. This includes using clear language and allowing enough patient time, as in every other indication. Comprehensive public education is crucial. We recommend enhancing methods for training physicians and educating patients to engage the entire population. Implementing these measures would surely enhance massive prophylaxis and prenatal care.

## Data Availability

The data presented in this study are available on request from the corresponding author due to ethical considerations related to the involved subjects.

## Abbreviations

CVS	chorionic villous sampling
DSD	disorders of sexual development
GC	genetic counseling
GW	gestational weeks
NIPT	non-invasive prenatal testing
OBG	obstetrician and gynecologist
SCAs	sex chromosomal aneuploidies
TOP	termination of pregnancy
RATs	rare autosomal anomalies
US	ultrasound
